# Impact of Habitual Flexion on Bone Formation After Spinal Fusion Surgery: An In Silico Study

**DOI:** 10.1002/jsp2.70075

**Published:** 2025-07-14

**Authors:** Siddarth Ananth Swaminathan, Nima Taheri, Luis Becker, Matthias Pumberger, Hendrik Schmidt, Sara Checa

**Affiliations:** ^1^ Julius Wolff Institute Berlin Institute of Health, Charité – Universitätsmedizin Berlin Berlin Germany; ^2^ Center for Musculoskeletal Surgery Charité– Universitätsmedizin Berlin Berlin Germany; ^3^ Institute of Biomechanics Hamburg University of Technology Hamburg Germany

**Keywords:** bone healing, finite element method, lumbar spine, spinal fusion

## Abstract

**Background:**

Lumbar spinal fusion is currently regarded as one of the most effective surgical treatments for patients with spinal deformities, degenerative disc disease, and degenerative spondylolisthesis. However, the procedure still faces a high incidence of non‐unions. A key factor contributing to non‐union is stress shielding effects related to unfavorable mechanical signals at the fusion site. Mechanical conditions at the fusion site are determined by the loading conditions that result from daily activities. Recent studies have reported that humans spend most of the day with their spine in a flexed position. The role of flexion loading in the progression of bone fusion remains poorly understood. This study explores the influence of habitual flexion loading on the spinal fusion process using a computational modeling framework that integrates finite element analysis with bone healing algorithms to simulate bone regeneration following fusion surgery.

**Methods:**

A finite element model of the lumbar spine based on a healthy subject was developed and validated with in vitro experimental data. Thereafter, a virtual posterior lumbar interbody fusion was performed where 2 intervertebral cages were inserted at the L4‐L5 level together with posterior fixation. The influence of two loading conditions on the predicted fusion process were investigated: (1) A compression load (2) A hybrid (compression + flexion) loading protocol simulating habitual flexion encountered during daily living.

**Results:**

Bone bridging was predicted to occur 14 weeks after surgery. At week 14, for the hybrid loading, the model predicted a bone volume of 70%, whereas for compression load, the bone volume prediction was 59%. Computer model predictions showed that habitual flexion loading can promote bone formation in the anterior and peripheral regions by inducing a mechanical environment favorable for bone formation.

**Conclusion:**

Flexion loading may enhance bone healing by promoting mechanically advantageous conditions. The computational framework could guide the development of optimized rehabilitation protocols to improve fusion outcomes.

## Graphical abstract

Lumbar spinal fusion is an effective treatment for spinal conditions, but non‐union remains a challenge due to unfavorable mechanical signals at the fusion site. This study used a computational model to investigate how habitual flexion loading affects bone fusion, finding that hybrid (compression + flexion) loading led to greater bone formation compared to compression alone. The results suggest that flexion loading may enhance bone healing, providing insights for optimizing rehabilitation protocols.

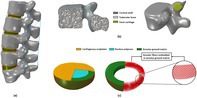

## 1 Introduction

Lumbar spinal fusion is currently recognized as one of the most efficient surgical interventions for patients presenting spinal deformities, degenerative disc diseases, and degenerative spondylolisthesis [1]. This procedure entails the fusion of two or more vertebrae to eliminate their relative mobility, typically accomplished through the utilization of a pedicle‐screw fixation system and/or the insertion of a spinal cage between the fused vertebrae [2, 3]. Intervertebral cages are instrumental in the biomechanical success of spinal fusion, as they maintain disc height during the process of bone regeneration [4]. However, non‐union rates ranging from 10% to 40% have been documented for mono‐segmental procedures, with higher incidence rates observed in multi‐segmental treatments [5, 6, 7].

One factor contributing to non‐union is stress‐shielding effects. This phenomenon involves improper load distribution between the implant and the surrounding tissue, which diminishes the mechanical stimuli necessary for bone formation, thereby impeding the fusion process [8, 9]. Given the significant challenge posed by non‐union rates in lumbar spinal fusion, particularly due to factors like stress‐shielding effects, researchers have increasingly turned to computational tools to better understand and predict fusion outcomes. Among these tools, finite element (FE) models have proven valuable in simulating the biomechanical conditions of the spine post‐surgery and can provide deeper insights into how various mechanical factors, including loading conditions, influence the success of the fusion process.

Computer models based on finite element analysis have been previously used to investigate the biomechanical conditions of the lumbar spine after fusion surgery [10, 11]. Furthermore, iterative computer models able to simulate the bone regeneration process have been used to investigate the role of mechanical signals [12], cage geometry [13] and surgical technique on the fusion outcome [14]. By leveraging mechano‐regulation algorithms for bone tissue formation, these models predict fusion outcomes under various conditions. However, existing models predominantly simulate compression loading, overlooking other biomechanical forces encountered during daily activities. Recent studies reveal that the human spine remains in a flexed position for much of the day [15], yet the contribution of flexion loading to bone regeneration remains unexplored.

Understanding the role of habitual flexion loading in spinal fusion has the potential to revolutionize both surgical planning and postoperative care. Insights into how flexion contributes to the mechanical environment of the fusion site could inform the selection of implants, such as intervertebral cages or pedicle screw systems, tailored to optimize load distribution. Furthermore, this knowledge could guide the development of patient‐specific rehabilitation protocols that encourage movements promoting optimal mechanical stimuli for bone regeneration, potentially reducing non‐union rates. By aligning surgical techniques and recovery strategies with biomechanical principles, the findings of this study could enhance long‐term outcomes for patients undergoing lumbar spinal fusion.

Therefore, the aim of this study was to investigate the contribution of habitual loading conditions to the spinal fusion process. Towards this aim, finite element computer models coupled with mechano‐regulated fusion algorithms were developed to investigate the role of habitual flexion on the spinal fusion outcome.

## 2 Materials and Methods

### 2.1 Physiological Model

#### 2.1.1 Model Geometry and Mesh

**FIGURE 1:**
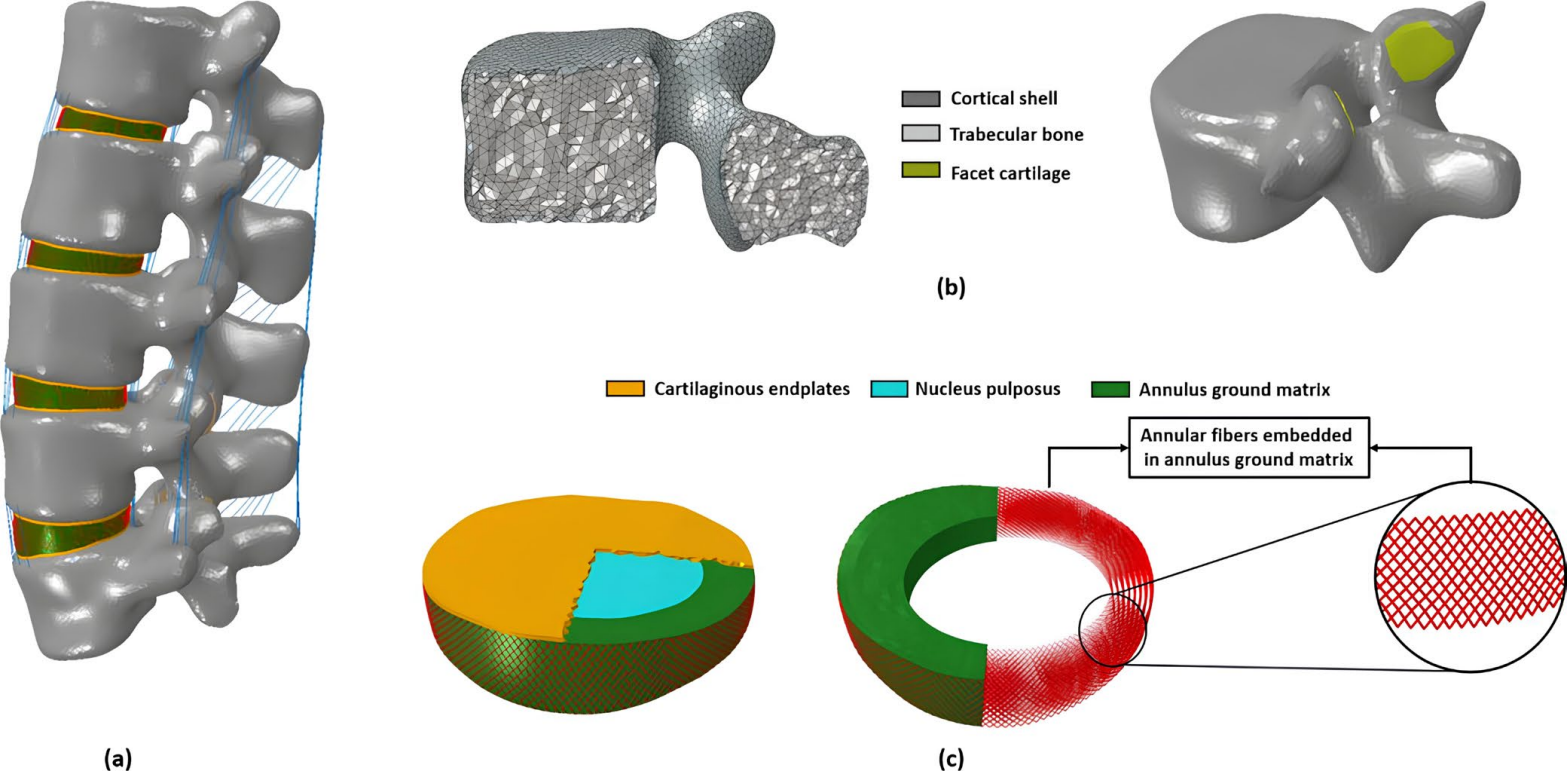
(a) Physiological finite element model of the L1—L5 lumbar spine (b) Geometry and volume mesh of the vertebra with cortical and trabecular bone (c) FE model of the intervertebral disc with cartilaginous endplates. The intervertebral disk was modeled as consisting of a nucleus pulposus and annulus ground matrix formed by eight layers of annular fibers.

A 3D non‐linear finite element model of an L1‐L5 lumbar spine was developed. The geometry was reconstructed based on CT scan data of a 50‐yearyear‐old male subject from the New Mexico Decedent Image Database [16]. The CT scans were imported to the commercial software Materialize Mimics 25.0 (Materialize, Leuven, Belgium) for segmentation, and 3D models of the vertebrae were developed. The 3D models were subsequently exported to Materialize 3‐matic 17.0 software. Here, local smoothing techniques were applied to eliminate any unwanted surface irregularities like spikes or cavities (Figure 1a).

The intervertebral disc was modeled consisting of the nucleus pulposus, occupying 45% of the disc volume [17], surrounded by the annulus ground matrix with fiber layers embedded in it. Additionally, the fibers were considered to exhibit a criss‐cross arrangement with alternating angles ranging from ±30° to ±45° [18] (Figure 1c). The annular fibers and the ligaments were modeled using non‐linear truss elements with only tensile properties. The facet cartilages were modeled according to the contour of the surface of the vertebra. The facet cartilages (Figure 1b) and the cartilaginous endplates were constructed with a thickness of 0.25 and 0.5 mm, respectively [19] (Figure 1).

Trabecular bone was incorporated by offsetting the outer surface of each vertebra by 1 mm with the outer layer representing the cortical shell [20, 21] (Figure 1b). The vertebrae were adaptively meshed with tetrahedral elements, with an average size of 1.5 mm. Further, the cartilaginous endplates and the intervertebral discs were meshed adaptively with an average size of 1 mm. For an optimum and efficient run time for the model, a mesh convergence test was performed for three different mesh sizes for the vertebra (2 mm, 1.5 mm, 1 mm), and two different sizes for the intervertebral disc and the cartilaginous endplates (0.5 mm, 1 mm). The mesh sizes for the physiological model were chosen with respect to the von mises stress developed in the different structures. The chosen mesh size resulted in von Mises differences lower than 5% in the vertebral body compared to the next finer mesh.

#### 2.1.2 Material Properties

Cortical, trabecular bone, cartilaginous endplates, nucleus pulposus, and the ground substance of the annulus fibrosus were modeled as linear elastic. The stiffness of annular fibers was modeled to progressively increase towards the inner layers [22, 23]. Furthermore, the fibers were modeled to occupy a volume fraction that varies from 23% (outermost) to 5% (innermost) within the annular ground substance [18, 24]. All seven major spinal ligaments (anterior longitudinal ligament (ALL), posterior longitudinal ligament (PLL), ligamentum flavum (LF), interspinous ligament (ISL), supraspinous ligament (SSL), intertransverse ligament (ITL), and capsular ligaments (CL)) were modeled as non‐linear with only tensile properties [25, 26]. The material properties of the various tissues are described in Table 1.

**TABLE 1:** Material properties of all the biological tissues in the FE model.

Tissue	Material model	E (MPa)	*Poisson's ratio*	Element type	Ref
Cortical shell	Isotropic homogeneous linear elastic	10 000	0.3	C3D4	[27]
Trabecular bone	Isotropic homogeneous linear elastic	100	0.3	C3D4	[27]
Cartilaginous endplates	Isotropic homogeneous linear elastic	23	0.4	C3D4	[28]
Facet cartilages	Isotropic homogeneous linear elastic	35	0.4	C3D4	[29]
Annular fibers	Non‐linear stress strain curves			T3D2	[23]
Ligaments	Non‐linear stress strain curves			T3D2	[26]
Intervertebral disc					
Annulus ground substance	Mooney‐Rivlin hyper elastic model	C1 = 0.18 MPa	C2 = 0.045 MPa	C3D8H	[30]
Nucleus pulposus	Mooney‐Rivlin hyper elastic model	C1 = 0.12 MPa	C2 = 0.03 MPa	C3D8H	[30]
Lumbar fusion—Bone healing					
Granulation	Isotropic homogeneous linear elastic	1	0.167	C3D4	[12]
Fibrous	Isotropic homogeneous linear elastic	2	0.167	C3D4	[31]
Cartilage	Isotropic homogeneous linear elastic	10	0.167	C3D4	[32]
Newly formed Bone	Isotropic homogeneous linear elastic	1000	0.325	C3D4	[12, 32]

#### 2.1.3 Loading and Boundary Conditions

The applied loading conditions aimed to replicate experimental in vitro protocols with the aim of performing a model evaluation. Two different loading conditions were simulated:

1. A pure moment of 7.5 Nm applied in all three anatomical directions [33].
2. A pure compression of 1000 N [34].

The moment was applied on the cranial surface of the L1 vertebra to simulate flexion, extension, lateral bending and rotation. The compression load was applied using the follower load technique where the load acts along the curvature of the spine [34]. The caudal surface of the L5 vertebra was fixed. Following previous studies, the interfaces between the endplates, disc and the vertebra were tied [35]. Surface‐surface contact behavior with a friction coefficient of 0.1 between the facet joints was used [14, 35].

#### 2.1.4 Evaluation of the Finite Element Model

For the evaluation of the finite element model, in vitro data and results from previous finite element models were used. In the case of pure bending, the predicted range of motion, load‐deflection behavior, and facet joint forces were compared with in vitro data [36, 37] and previous FE studies [38]. For a pure compressive force, the predicted L4‐L5 nucleus pulposus pressure was compared with those previously reported in in vitro [39] and finite element studies [38].

#### 2.1.5 Evaluation of the Finite Element Model Post Ligament Resection

During surgical fusion, the ligaments and facets are resected. Consequently, additional analysis was conducted by resecting the ligaments and facets of the L4‐L5 motion segment and comparing the resulting range of motion with data from an in vitro experiment [40]. The L4‐L5 segment was isolated, and the ligaments were sequentially resected to develop models representing various states: without the thoracolumbar ligament (NoTL), supraspinous ligament (NoSLL), interspinous ligament (NoISL), ligamentum flavum (NoLF), facets (NoFacet), posterior longitudinal ligament (NoPLL), and anterior longitudinal ligament (NoALL). These models were subjected to a simulated pure moment loading of 10 Nm, and their range of motion was compared to experimental results.

### 2.2 Post‐Surgical Model

#### 2.2.1 Model Geometry

**FIGURE 2:**
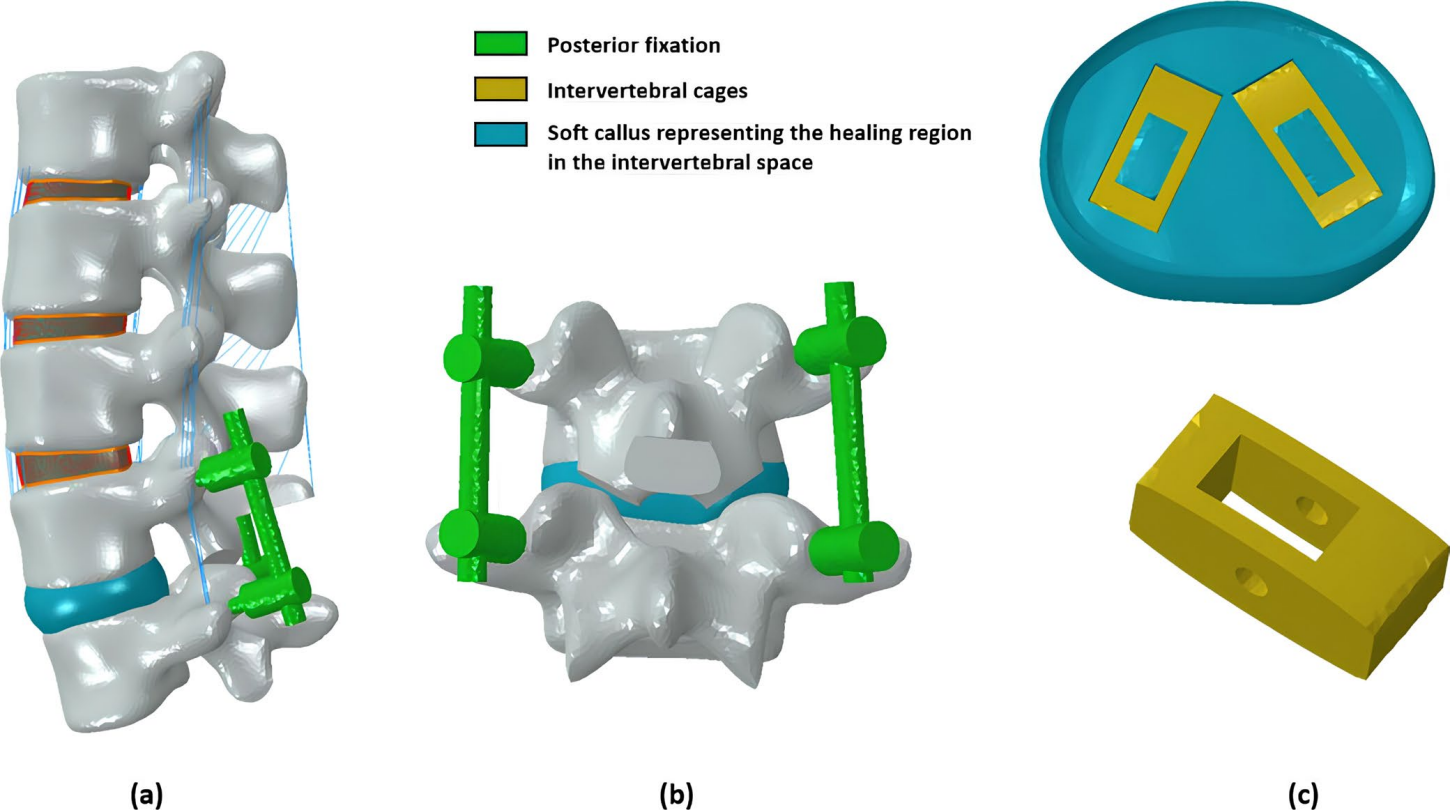
(a) Sagittal view of the post‐surgical finite element model and (b) L4‐L5 posterior view with the posterior fixation system and (c) Two intervertebral cages within the fusion region.

To simulate the fusion surgery, a virtual Posterior Lumbar Interbody Fusion (PLIF) was performed at the L4–L5 level by removing the facet joints from the L4–L5 vertebrae, a part of the spinous process of the L4 vertebra and removing the ligaments except the ITLs (Figure 2a). The IVD at the L4‐L5 level was removed and two solid cages with 9 and 10 mm height and width, respectively, were virtually implanted. Furthermore, an additional fixation device consisting of two sets of screws and rods was placed on the vertebrae posteriorly to provide additional stability (Figure 2b). Additionally, to represent the post‐operative condition, a soft callus representing the healing region was modeled (Figure 2c). The intervertebral cages, fixation device, and the soft callus were adaptively meshed with an average mesh size of 1 mm with C3D4 elements. The implants and the vertebra contact were modelled with tie constraints [41].

#### 2.2.2 Material Properties

The cages were considered to be made of PEEK (Young's modulus: 3.5 GPa, Poisson's ration:0.3) or Titanium (Ti) (Young's modulus: 110 GPa, Poisson's ratio: 0.3) [42, 43]. The posterior fixation was considered to be made of Titanium (Young's modulus: 110 GPa, Poisson's ratio 0.3) [42]. The callus region was initially modeled as granulation tissue (1 MPa) to simulate the early healing state [12].

#### 2.2.3 Simulation of the Lumbar Fusion Process

**FIGURE 3:**
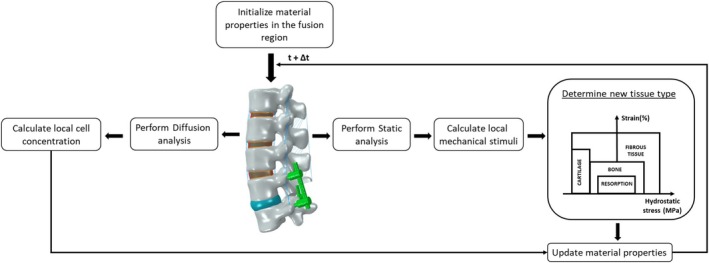
Flowchart to simulate spinal fusion using the mechano‐differentiation algorithm of Claes and Heigele et al. 1999.

An iterative algorithm was implemented to simulate the temporal formation of bone within the fusion region. Based on previous studies, bone formation was assumed to be regulated by the mechanical environment within the fusion region [12, 13, 32]. The algorithm is based on an iterative process, where, at each iteration, the mechanical conditions within the fusion region determine the formation of a specific tissue phenotype (Figure 3). This change in tissue phenotype is then implemented as a change in the element material properties (Table 1) of the finite element model.

Initially, it was assumed that the entire fusion region was filled with granulation tissue. The migration of cells towards the fusion region was modeled as a diffusive process governed by equation (1), with the vertebral endplates acting as the cell source.

(1) $$\mathrm{dn}/\mathrm{dt}=D\nabla^{2}n$$

where the cell density n is determined from the diffusion coefficient D. The cell density was normalized between 0 and 1. The diffusion coefficient was determined such that the cell coverage reaches the entire intervertebral space within the first 4 weeks, with a maximum cell density of 1 [13].

In each iteration, the mechanical stimulus (minimum principle strains and the hydrostatic pressure) was calculated according to the mechano‐regulation rules for tissue formation [12, 44]. The resorption limits used to simulate bone resorption are 0.1% and 0.15 MPa for minimum principal strains and hydrostatic pressure, respectively [12].

Bone fusion was simulated for 15 iterations (~105 days), where 1 iteration is equivalent to 1 week. The FE software ABAQUS/Standard 2021 (Simulia, Dassault Systèmes) was used for the finite element simulations, and the Python programming language was used to implement the iterative fusion process.

#### 2.2.4 Loading Conditions

Two different loading conditions were analyzed to investigate the effect of mechanical loading on the bone formation patterns.

1. A compressive force of 500 N was applied, similar to previous studies [12, 13].
2. The hybrid loading condition, simulating habitual flexion, combined flexion and compression based on studies showing the lumbar spine is flexed for approximately 63% of daily activities (about 15.1 h), typically between 10° and 40° [16]. To reflect this, 63% of the load was applied as compression plus flexion, and 37% as pure compression, representing a realistic daily loading distribution. For the hybrid loading protocol, the range of motion is assumed to be the same before and after the fusion surgery based on the premise that individuals tend to bend their spines in similar manners irrespective of their surgical or healthy state [45, 46]. Therefore, a moment load of 7.5 Nm [47] under a constant compressive load of 500 N [48] was applied to the physiological model and the ROM was obtained which was 19.37 degrees. For the post‐surgical model, the moment load was incrementally increased keeping the compressive pre‐load constant until the post‐surgical model reached the same ROM as the physiological model.

## 3 Results

### 3.1 Evaluation of the Physiological Model

**FIGURE 4:**
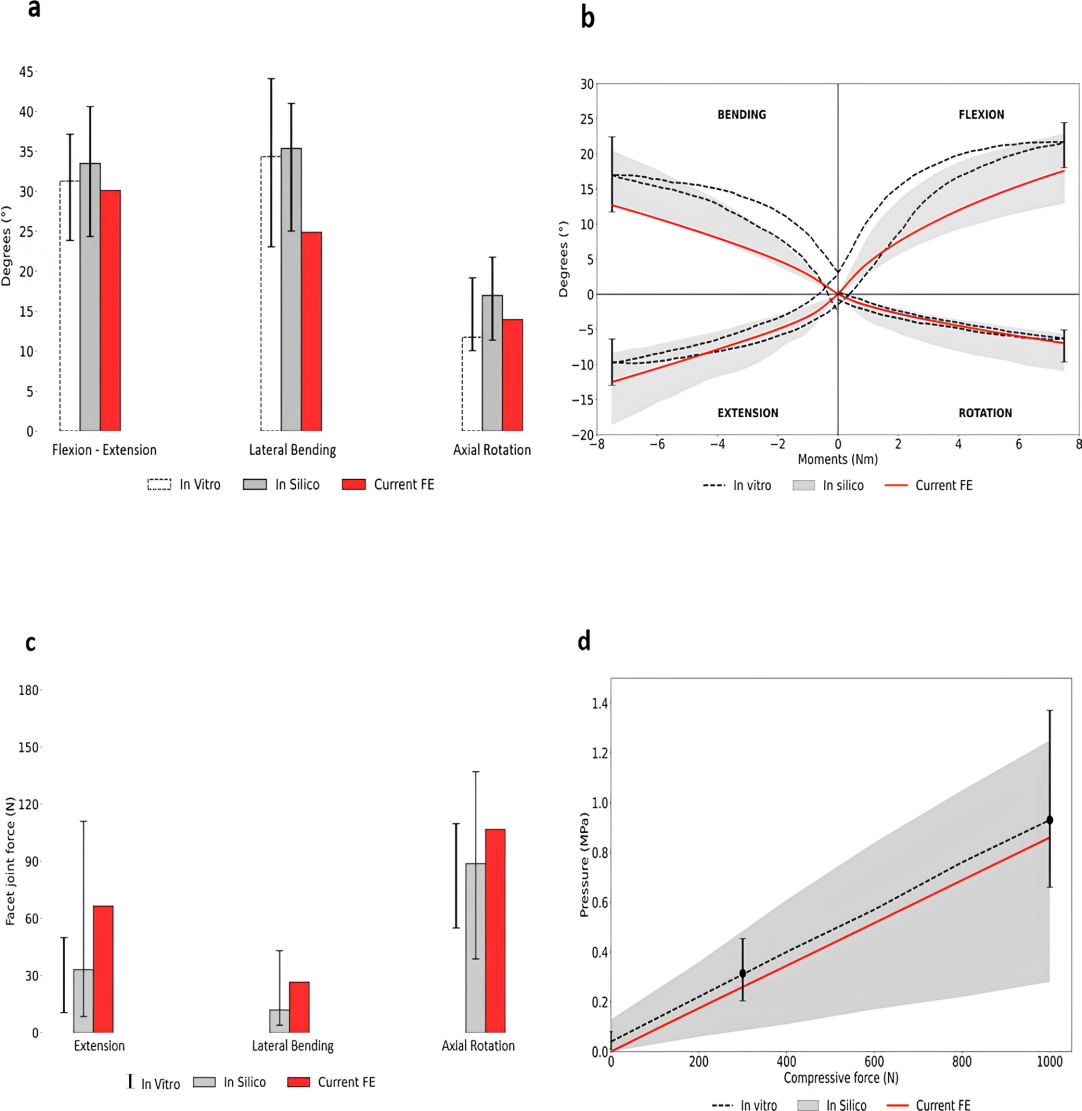
(a) Predicted total range of motion values in flexion‐extension, lateral bending, and axial rotation. The white dotted bars and their ranges correspond to the median and the range of in vitro measurements [37]. The gray bars and their range represent the median and the range of several published FE results [38]. (b) Load‐deflection curve: The median results of in vitro experiments are shown with a black dashed line, the black error bars represent the range of results at 7.5 Nm [37]. The ranges of the in silico results are shown in gray bands [38]. (c) The mean facet joint force values at all spinal levels. The black error bars shows the range of facet joint forces measured in vitro [36]. The gray bars and their ranges show the median, the minimum, and maximum values of in silico results [38]. (d) Intradiscal pressure values of the nucleus pulposus at L4–L5 at 1000 N compressive follower load. The black dashed line shows the median result of an in vitro measurement, while the black error bars represent the minimum and maximum values for 0, 300, and 1000 N [39]. The in silico results are shown as a gray band [38].

A total L1–L5 range of motion in flexion‐extension, lateral bending, and axial rotation of 30°, 25°, and 14° was predicted, respectively. This range of motion is within the range of experimental in vitro data [37] and the range predicted by other in silico studies [38] (Figure 4a).

Furthermore, the nonlinear load‐deflection curves illustrate the predicted stiffening of the lumbar spine against moment loads of up to 7.5 Nm (Figure 4b). For all motions, model predictions were within the range reported in previous experimental and numerical studies.

The mean facet joint force values were 66.5, 26.56, and 106.7 N in extension, lateral bending, and axial rotation, respectively. When compared with in vitro data [36], model predictions agree well for all the motions except for extension, where the finite element model slightly overpredicted the forces. The model predictions are well within the ranges of other in silico studies [38] (Figure 4c).

The intradiscal pressure value under a compressive follower load of 1000 N was 0.85 MPa. This value is in good agreement with both previous experimental [39] and in silico [38] studies (Figure 4d).

### 3.2 Evaluation of the Finite Element Model Post Ligament Resection

**FIGURE 5:**
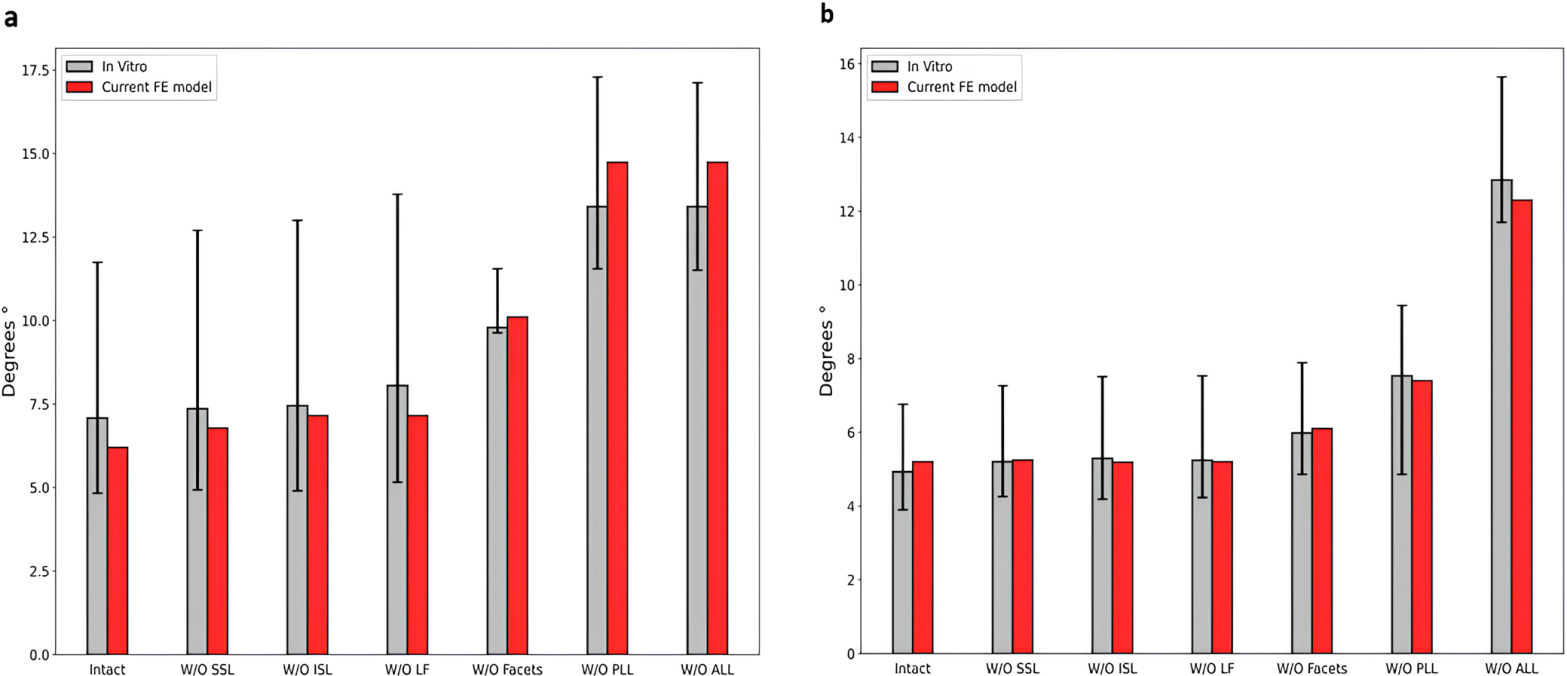
Predicted range of motion values for applied moment of 10 Nm with stepwise reduction of ligaments from the posterior region (a) Flexion (b) Extension.

The range of motion of the isolated L4‐L5 segment of the physiological model after resection of the ligaments from the posterior region was compared with the experimental data [40] for flexion and extension. For both motions, finite element model predictions fall within the range of reported experimental values (Figure 5).

### 3.3 Habitual Flexion Influences the Predicted Bone Fusion Process

**FIGURE 6:**
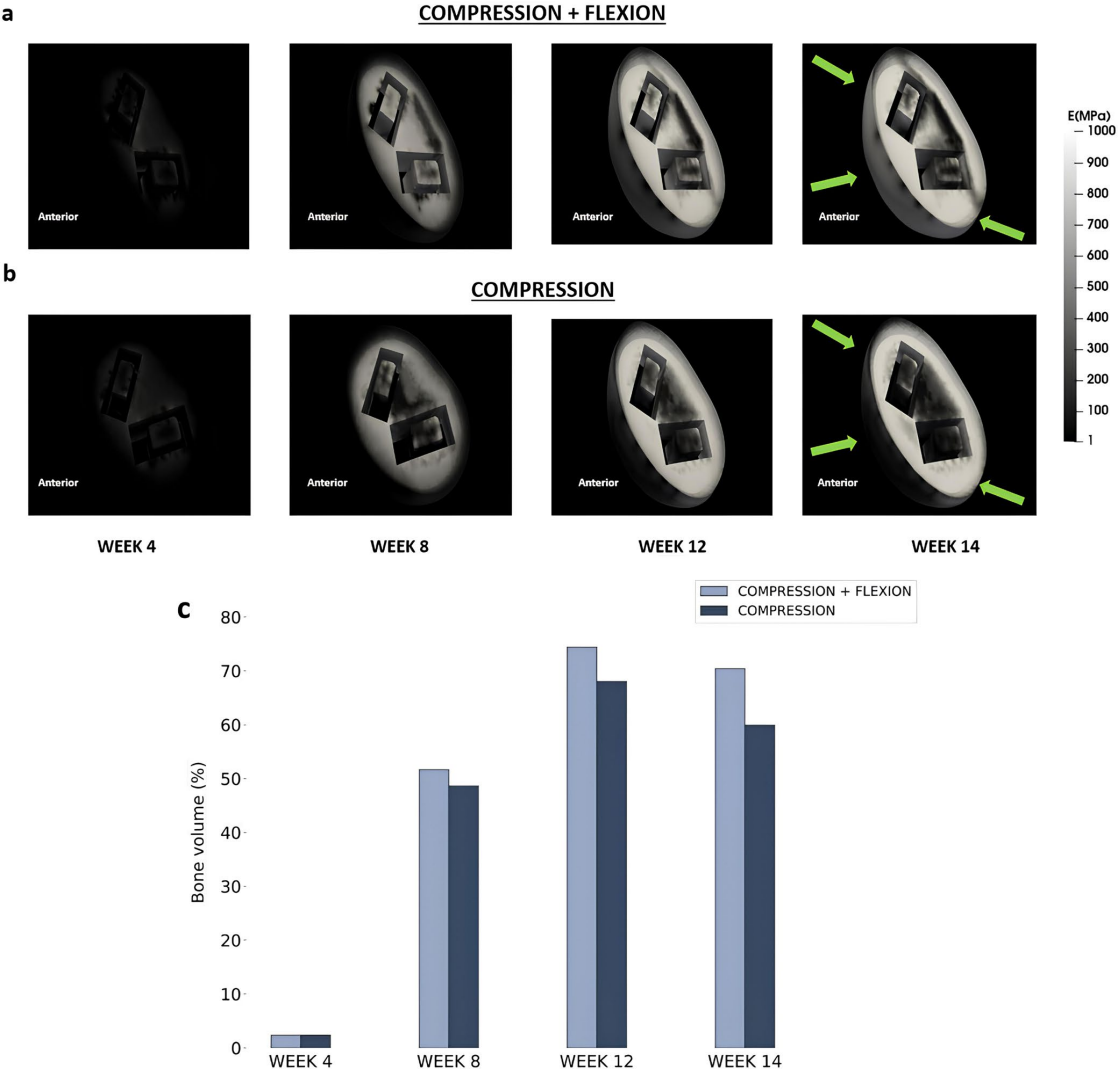
Predicted fusion patterns for Ti cage (a) compression + flexion (b) compression alone. The green arrows show the anterior and peripheral regions in both loading conditions indicating the difference in bone formation (c) Bone volume % in the intervertebral space at weeks 4, 8, 12 and 14 for both loading conditions.

The predicted fusion pattern was influenced by the type of mechanical loading. Pure compression and combined compression and flexion led to different bone tissue formation patterns over the course of bone formation. For both loading conditions, the bone formation was predicted to start after around week 4, and fusion was predicted between week 8 and week 12. Until week 8, predicted bone formation was similar for both loading conditions, as the bone formation started near the surface of the vertebrae and progressed towards the central region of the intervertebral space (Figure 6).

After week 8, bone formation was predicted in the anterior and peripheral regions under combined compression and flexion loading, and early stages of bony bridging were predicted at the caudal and cranial surfaces of the L4 and L5 vertebral bodies, respectively (Figure 6a). At week 14, an area of matured bone formed in the anterior and the peripheral regions bridging the space between the vertebral bodies. Moreover, inside the cage, bone with material properties similar to those of cancellous bone was predicted.

Under pure compression, after 8 weeks, bone formation was predicted all around the cages, with little bone formation predicted in the region within or between the cages. Over the healing process, previously formed bone within and in between the cages was slowly resorbed (Figure 6b). The amount of predicted bone formation during the whole fusion process was lower under pure compression when compared with combined compression and flexion loading (Figure 6c).

**FIGURE 7:**
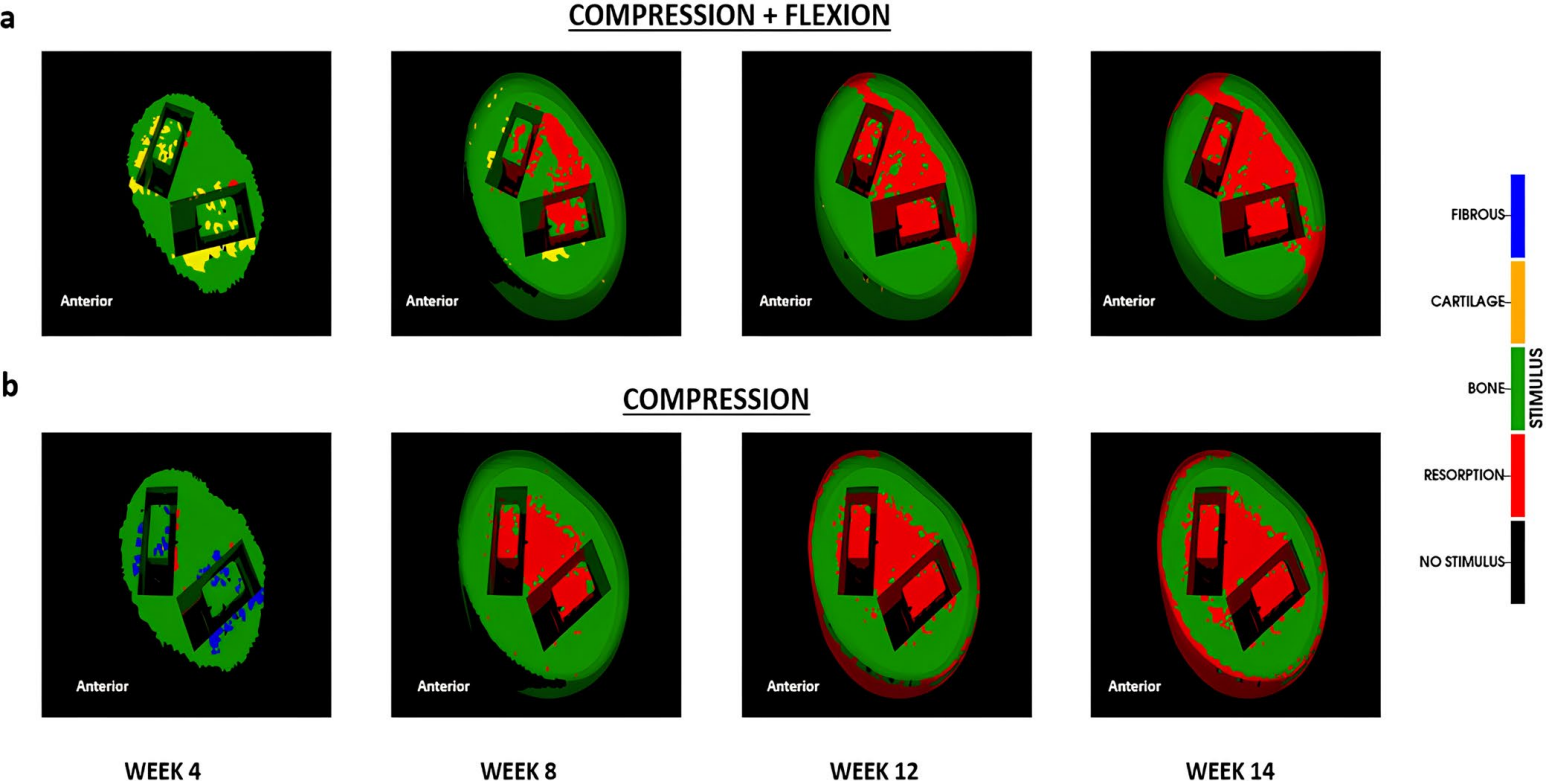
Mechanical stimulus according to the mechano‐differentiation algorithm (a) compression + flexion (b) compression alone.

In terms of the biomechanical conditions within the fusion region, under both loading conditions, most of the fusion region was predicted to be initially under mechanical stimuli favorable for bone formation, with some small regions around the cage favorable for cartilage or fibrous tissue formation. At week 8, under both loading conditions, the space within and between the cages showed stress shielding effects, where a mechanical environment favorable for bone resorption was predicted. However, the resorption region was smaller under combined compression and flexion load. Moreover, under pure compression, resorption was predicted within the anterior peripheral region. In addition, mechanical loading influenced the temporal change of the distribution of mechanical stimuli within the fusion region. While under pure compression, the distribution of mechanical stimuli within the fusion region did not considerably change over the healing process, under combined compression and flexion, mechanical stimuli favorable for bone formation shifted to the anterior and peripheral regions as the fusion process progressed (Figure 7).

### 3.4 Cage Material Did Not Influence Fusion Patterns

**FIGURE 8:**
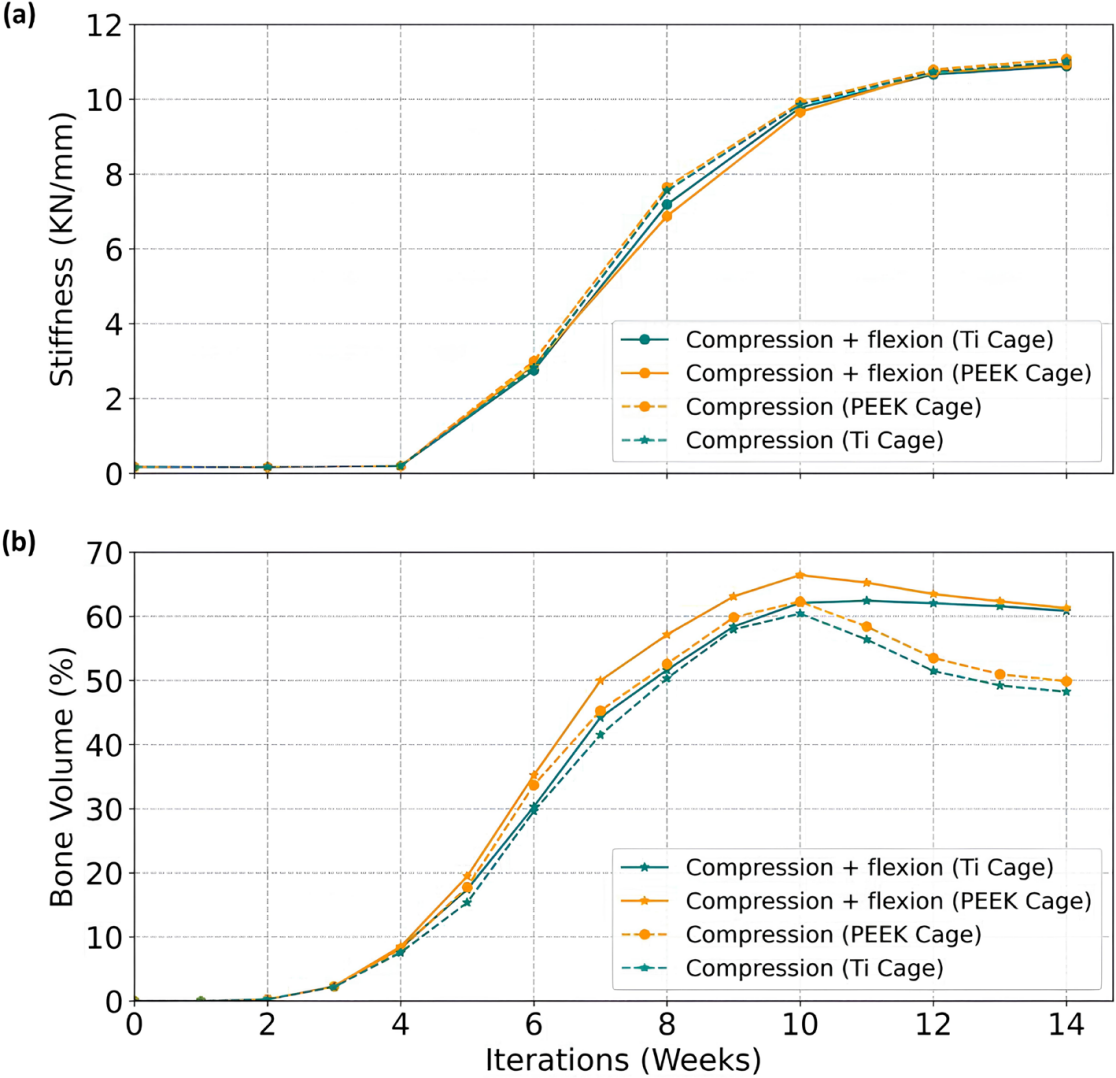
Temporal changes in (a) Stiffness without the contribution of the implants (b) Bone volume (%). The dashed lines represent the compression load and the solid lines represent the compression + flexion load.

For both titanium and PEEK cages, the predicted fusion patterns were similar for both loading conditions. The predicted compressive stiffness for both cage types was similar over the fusion process for both loading conditions (Figure 8a); with peak compressive stiffness at week 14.

In terms of bone volume, a PEEK cage resulted in slightly higher bone formation under combined compression and flexion during the fusion process compared to a titanium cage. However, at week 14, both cage materials resulted in very similar bone formation volumes (Figure 8).

**FIGURE 9:**
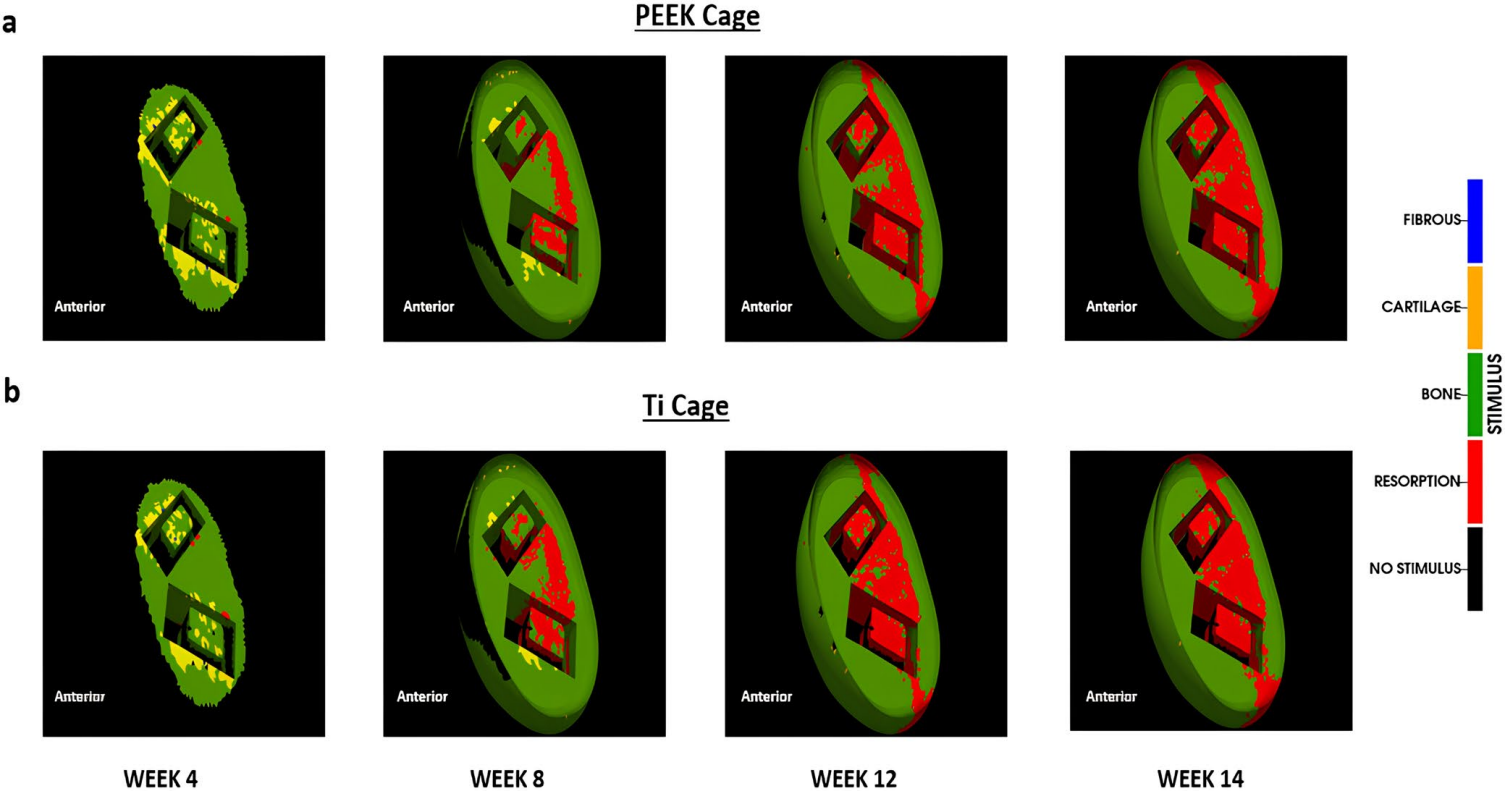
Mechanical stimulus according to the mechano‐differentiation algorithm for compression + flexion load (a) PEEK cage (b) Ti cage.

In terms of mechanical stimulus developed in the fusion region, both the PEEK and the Ti cage resulted in a similar environment for bone formation until week 8. Furthermore, after week 8, the mechanical environment in the regions inside the cage falls within the resorption stimulus, resulting in bone resorption and subsequent bone removal (Figure 9).

**FIGURE 10:**
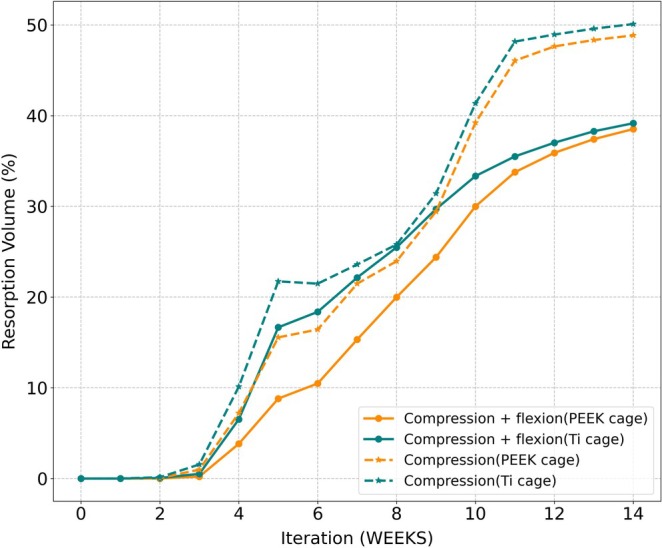
Temporal changes in volume (%) of regions under a resorption mechanical stimulus for Ti and PEEK cage. Solid line representing compression + flexion load and dashed line representing compression load.

The resorption zone volume increases over time across all conditions, with higher values observed under the combined compression and flexion loading compared to compression alone. Notably, the Ti cages showed greater resorption volumes than PEEK cages under both loading scenarios (Figure 10). This suggests that the stiffer Ti material may contribute to increased stress shielding, leading to enhanced bone resorption.

## 4 Discussion

Lumbar spinal fusion is a very complex biological process where mechanical stimuli serve as a potent driver for bone formation [12]. These mechanical stimuli are, among other factors, largely dictated by the mechanical loads occurring in the spine. Recently, it has been reported that spinal flexion is the most dominant movement, as humans keep their spine flexed between 0° and 50°94% of the time in a day [15]. Moreover, it has been reported that individuals try to bend their spine regardless of whether their spine is healthy or has undergone surgical intervention [47, 49]. Despite recognition of the role of mechanical loading in spinal fusion, the influence of habitual spinal flexion on bone fusion patterns remains largely unknown. This study examines the effects of mechanical loading conditions on lumbar spinal fusion, demonstrating that habitual spinal flexion facilitates bone formation progression and creates a more dynamic mechanical environment within the fusion region.

The finite element model of the post‐surgical condition was built based on a finite element model of the physiological state. The predicted biomechanics of the physiological finite element model was compared with in vitro data [38] and other published in silico models of the lumbar spine. The model agreed with the in vitro and the in silico results for flexion‐extension, lateral bending, and axial rotation. However, the finite element model predicted slightly lower flexion stiffness when compared with previous in vitro data. This trend has also been reported by previous finite element models [38]. Predicted facet joint forces were slightly overpredicted in extension, while they compared well with experimental data in lateral bending and axial rotation. This overprediction can be due to the simplified geometry of the facet cartilages as they were not seen in the CT scans [50, 51].

The model was developed for a healthy individual. In the future, the model could be adapted to represent other patient groups, by changing for example the geometry and/or the material properties.

In this study, a mechano‐regulation algorithm was used to predict bone formation after lumbar fusion surgery and to investigate the effect of habitual flexion on the healing outcome. Under combined compression and flexion load, predicted bone formation patterns were higher in the anterior and peripheral regions compared with isolated compression. This can be attributed to habitual flexion resulting in a mechanical stimulus within the anterior and peripheral regions which, according to the current state of the art on the mechano‐regulation of bone fusion, appears beneficial to bone formation. The predicted bone formation patterns at early stages of fusion are in accordance with clinical observations [52] showing early bone formation mainly outside the cages. However, at later stages, for combined compression and flexion load, formation of bony arthrodesis with tissue properties similar to cancellous bone within the cages was predicted, similar to findings reported in clinical studies [52].

The strain and hydrostatic pressure thresholds used were adopted from long bone healing studies, for which the thresholds have been extensively studied and evaluated with clinical studies. The investigation of the mechanobiology of bone regeneration in the context of spinal fusion is rather limited, with only very few studies looking into the effect of mechanical signals on spinal bone regeneration [12]. Previous studies have shown that the application of the differentiation algorithms from long bone healing are able to explain the progression of spinal fusion [13, 14], however a detailed validation has not been performed so far. This can be explained by the lack of clinical data of the follow up of fusion processes. Clinically, spinal fusion is usually evaluated using radiological images after 3 or 6 months, which does not allow a rigorous validation. There is a need to further validate these thresholds in the spinal fusion context. Future studies should aim to clinically evaluate the dynamics of the fusion process. This would allow a better comparison with model predictions and thereof, the validation of these thresholds.

The finding that habitual flexion may promote fusion suggests potential implications for postoperative care. Controlled flexion could offer beneficial mechanical stimuli, informing rehabilitation protocols or activity guidelines. However, further clinical validation is needed before translating these insights into practice.

In our model, most spinal ligaments were removed to reflect the typical surgical procedure of posterior lumbar interbody fusion (PLIF), where substantial resection of soft tissues is performed to access the disc space and decompress neural structures. This modeling choice aligns with previous computational studies simulating PLIF [53, 54] and with clinical practices [55]. A preliminary study of the effect of the ligaments on the fusion response showed no considerable influence of the ligaments on the predicted healing outcome (Supplementary). This can be explained by the fact that the spinal cages and the fixation are stiffer than the ligaments, therefore defining the mechanical conditions within the healing region.

Further, a parametric analysis of the influence of tissue material properties on the predicted range of motion and disc pressure in the physiological model showed little effects to changes of bone material properties. Moreover, fusion predictions were not sensitive to differences in bone or intervertebral disc material properties reported in the literature [12, 56] (Supplementary).

Previous studies have reported that fusion cages made of titanium or PEEK do not lead to different fusion outcomes [57, 58, 59, 60, 61, 62]. Our study agrees with those observations, where irrespective of the cage material, no considerable difference in formed bone volume was predicted.

In this study, we predicted that the tested fusion cages can lead to considerable stress shielding effects within the cages and in the region between the two cages. This effect was predicted to be slightly reduced under habitual flexion, specifically during the early fusion stages. Stress shielding has been reported in many clinical fusion studies [63, 64] and it is one of the main motivations for the use of bone graft material within the interior of the cages [65, 66] or the use of a bone stimulator, e.g., BMP [67, 68, 69].

Although this study used a hybrid loading condition based on 24‐hour sagittal movement of a human to simulate lumbar fusion, the framework could be extended to patient‐specific applications. Wearable sensor or motion capture data could be used to define loading profiles, supporting personalized rehabilitation planning and outcome prediction.

### 4.1 This Study Has Some Limitations That Merit Consideration

The vertebral body material properties were assumed to be isotropic, whereas the bone is anisotropic in nature. However, it has been reported that the material properties of the bony structures have a very low impact on the spinal kinematics [70, 71].

The model simplifies the implant‐bone interaction with a tie constraint. While this simplification facilitates numerical stability and is consistent with prior studies [41, 72], it does not fully capture the micromotion or potential sliding that can occur during healing periods.

In addition, the analysis was performed with simplified loading conditions—a follower load simulating compression and a moment load to simulate flexion. It should be noted that this simplification does not fully replicate the complexity of real‐world loading conditions. Factors such as dynamic variations in spinal posture, individual differences in daily activity patterns, and potential deviations from the assumed flexion range may influence the mechanical environment experienced during habitual activities.

Due to the lack of quantitative clinical data on post‐operative spinal loading and patient‐specific motion patterns during recovery, a constant range of motion was assumed throughout the healing period. These limitations are acknowledged, and future studies could refine the loading model to incorporate a broader range of dynamic, individualized, and posture‐dependent loading conditions.

While this study focused on the influence of loading conditions and cage material properties, future studies could incorporate parametric analyses to systematically investigate geometrical parameters, cage spacing and angulation on bone formation.

The simulation of the fusion process was idealized, where cell migration was modeled as a diffusive process neglecting cell mitosis and apoptosis while keeping the diffusion coefficient constant. The mechano‐regulation of bone formation during the fusion process is based on theories initially developed for long bone healing; however, previous in silico studies have shown the potential of these rules for the prediction of fusion processes in the spine [12, 13]. The model assumed that the material properties of the structures surrounding the fusion region remained unchanged during the fusion process. Clinically, changes in the adjacent structures have been reported, which often lead to the degeneration of the adjacent segments. The contribution of those changes to the fusion process and the role of the mechanical environment on the progression of tissue degeneration in adjacent structures remain to be investigated. The temporal progression of bony fusion in this study is represented in weeks, consistent with the bone healing algorithm parameters previously employed to simulate lumbar fusion. However, the time scale requires further validation due to the limited availability of clinical data.

The 15‐week simulation period reflects a clinically relevant timeframe corresponding to early postoperative recovery after lumbar fusion, during which initial signs of fusion and functional improvement are typically assessed [73, 74]. While individual healing rates vary due to factors like patient biology, surgical technique, and rehabilitation, modelling this variability was beyond the scope of this study.

This study did not model the effects of bone grafts or osteo‐inductive agents such as BMPs, which can accelerate fusion and alter healing patterns. Future work could approximate their influence by adjusting biological parameters like cell density or proliferation rates to reflect enhanced clinical conditions.

With the above limitations taken into consideration, future work will focus on integrating patient‐specific kinematic data to more accurately capture daily activity patterns. The model may also be extended to predict adjacent vertebral and intervertebral disc adaptations to the fusion surgery by incorporating bone and disc remodelling algorithms, respectively. These additions will enable more comprehensive simulations and might allow the future use of the model to inform rehabilitation protocols.

In conclusion, a computer model of an L1–L5 lumbar spine was developed and a PLIF was virtually simulated to investigate the contribution of habitual flexion loading to the bone fusion process. The model predicts increased bone formation for habitual flexion compared with pure compression loads. The computational framework could in the future be used to develop rehabilitation protocols that support the healing outcome.

## Conflicts of Interest

The authors declare no conflicts of interest.

## Supporting information


**Data S1** Supporting Information.
