# Supplementary material for: Impact of Habitual Flexion on Bone Formation After Spinal Fusion Surgery: An In Silico Study
Source: JOR Spine. 2025 Jul 14;8(3):e70075. doi: 10.1002/jsp2.70075 (PMC12258091; doi:10.1002/jsp2.70075)
Supplement: Supplementary file 1 — Data S1 Supporting Information. [file JSP2-8-e70075-s001.docx]

**SUPPLEMENTARY DATA**

**IMPACT OF RESECTION OF LIGAMENTS ON THE FUSION**

Most spinal ligaments were removed in the model to reflect standard PLIF procedures, consistent with prior computational and clinical pratices [1-3] . A separate analysis with intact ligaments in the fusion region showed no impact on bone regeneration, likely because the stiffer cages and fixation systems dominate the mechanical environment during healing.


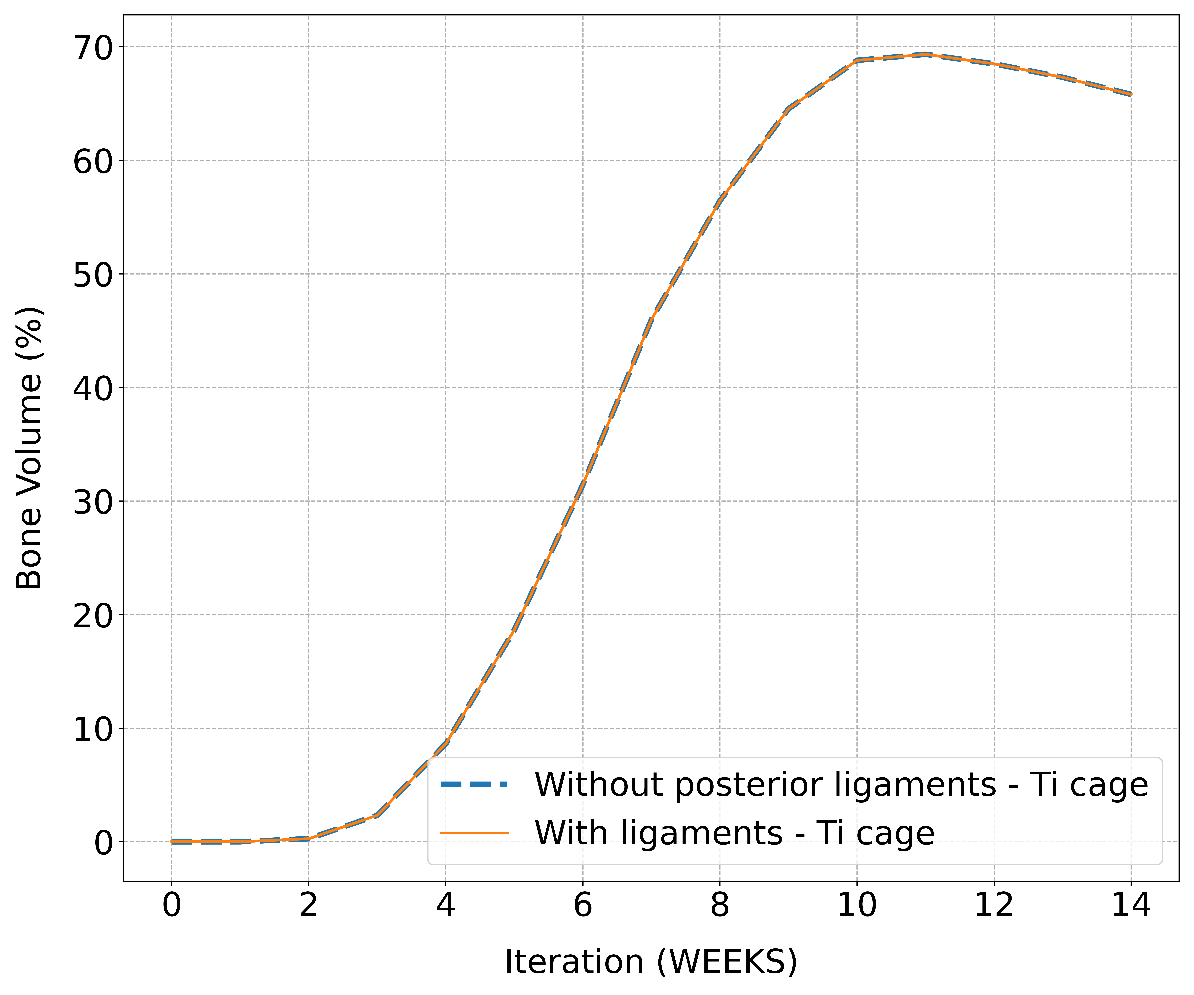


**MATERIAL SENSITIVITY ON FUSION**

An additional analysis was conducted to assess the influence of stiffer bone and intervertebral disc material properties, using values from the literature [4, 5]. The predicted bone formation during the fusion process remained unchanged, indicating minimal sensitivity to these properties (see Table and Figure below).

**Table 1. Softer Material properties**

| **Tissue** | **E (MPa)** | ***v* (Poisson’s ration)** | **Ref** |
| --- | --- | --- | --- |
| Cortical bone | 10000 | 0.3 | [5] |
| Trabecular bone | 100 | 0.3 |  |
| **Intervertebral disc** | | | |
|  | **C1 (MPa)** | **C2 (MPa)** | [6] |
| Annulus Fibrosus | 0.18 | 0.045 |  |
| Nucleus pulposus | 0.12 | 0.03 |  |

**Table 2. Stiffer Material properties**

| **Tissue** | **E (MPa)** | ***v* (Poisson’s ration)** | **Ref** |
| --- | --- | --- | --- |
| Cortical bone | 15750 | 0.3 | [5] |
| Trabecular bone | 400 | 0.3 |  |
| **Intervertebral disc** | | | |
|  | **C1 (MPa)** | **C2 (MPa)** | [4] |
| Annulus Fibrosus | 0.56 | 0.14 |  |
| Nucleus pulposus | 0.12 | 0.09 |  |


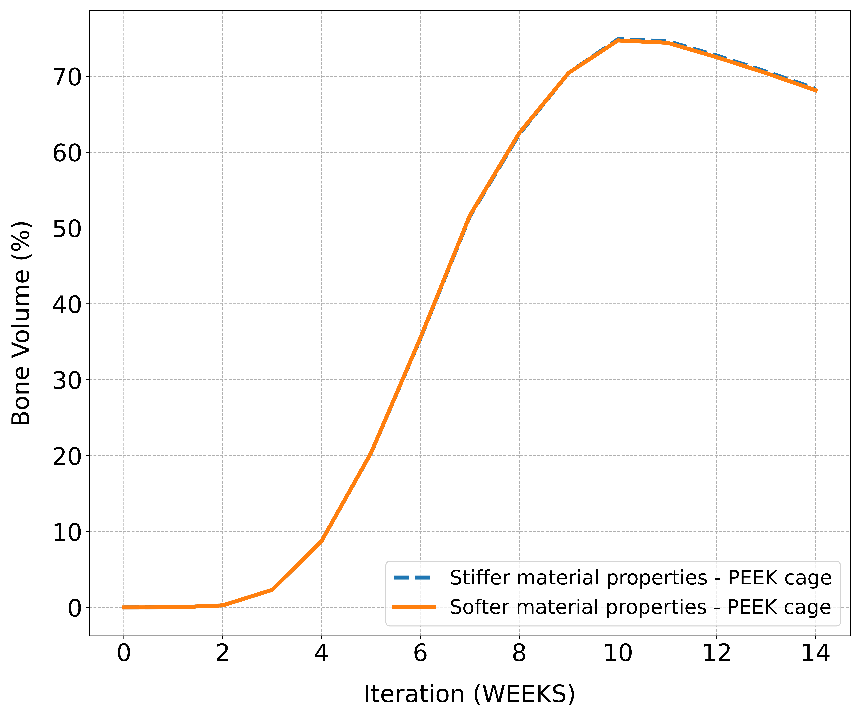


**References**

1. Tsuang, Y.-H., et al., *Comparison of cage application modality in posterior lumbar interbody fusion with posterior instrumentation—A finite element study.* Medical engineering & physics, 2009. **31**(5): p. 565-570.

2. Umale, S., et al., *A biomechanical investigation of lumbar interbody fusion techniques.* Journal of the mechanical behavior of biomedical materials, 2022. **125**: p. 104961.

3. Mobbs, R.J., et al., *Lumbar interbody fusion: techniques, indications and comparison of interbody fusion options including PLIF, TLIF, MI-TLIF, OLIF/ATP, LLIF and ALIF.* Journal of spine surgery, 2015. **1**(1): p. 2.

4. Xiao, Z., et al., *Biomechanical evaluation of three surgical scenarios of posterior lumbar interbody fusion by finite element analysis.* Biomedical engineering online, 2012. **11**: p. 1-11.

5. Bashkuev, M., et al., *Computational analyses of different intervertebral cages for lumbar spinal fusion.* Journal of Biomechanics, 2015. **48**(12): p. 3274-3282.

6. Zhang, C., et al., *Does Osteoporosis Affect the Adjacent Segments Following Anterior Lumbar Interbody Fusion? A Finite Element Study.* World Neurosurgery, 2021. **146**: p. e739-e746.
